# Feeding strategies for the acquisition of high‐quality food sources in stream macroinvertebrates: Collecting, integrating, and mixed feeding

**DOI:** 10.1002/lno.10818

**Published:** 2018-05-06

**Authors:** Fen Guo, Stuart E. Bunn, Michael T. Brett, Brian Fry, Hannes Hager, Xiaoguang Ouyang, Martin J. Kainz

**Affiliations:** ^1^ WasserCluster Lunz—Inter‐University Centre for Aquatic Ecosystem Research Lunz am See Austria; ^2^ State Key Laboratory of Environmental Criteria and Risk Assessment Chinese Research Academy of Environmental Sciences Beijing China; ^3^ Australian Rivers Institute, Griffith University Nathan Queensland Australia; ^4^ Department of Civil and Environmental Engineering University of Washington Seattle Washington; ^5^ Simon F.S. Li Marine Science Laboratory, School of Life Sciences, and Earth System Science Programme The Chinese University of Hong Kong Hong Kong China

## Abstract

Aquatic macroinvertebrates play an important functional role in energy transfer in food webs, linking basal food sources to upper trophic levels that include fish, birds, and humans. However, the trophic coupling of nutritional quality between macroinvertebrates and their food sources is still poorly understood. We conducted a field study in subalpine streams in Austria to investigate how the nutritional quality (measured by long‐chain polyunsaturated fatty acids, LC‐PUFAs) in macroinvertebrates changes relative to their basal food sources. Samples of macroinvertebrates, periphyton, and leaves were collected from 17 streams in July and October 2016 and their fatty acid (FA) composition was analyzed. Periphyton FA varied strongly with time and space, and their trophic effect on macroinvertebrate FA differed among functional feeding groups. The match between periphyton FA and macroinvertebrate FA decreased with increasing trophic levels, but LC‐PUFA content increased with each trophic step from periphyton to grazers and finally predators. Macroinvertebrates fed selectively on, assimilated, and/or actively controlled their LC‐PUFA, especially eicosapentaenoic acid (EPA, 20 : 5ω3) relative to their basal food sources in the face of spatial and temporal changes. Grazer FA profiles reflected periphyton FA with relatively good fidelity, and especially their EPA feeding strategy was primarily linked to periphyton FA variation across seasons. In contrast, shredders appeared to preferentially assimilate more EPA over other FA, which was determined by the availability of high‐quality food over seasons. Predators may more actively control their LC‐PUFA distribution with respect to different quality foods and showed less fidelity to the basal FA profiles in plants and prey. Overall, grazers and shredders showed relatively good fidelity to food FA profiles and performed as both “collectors” and “integrators” for LC‐PUFA requirements across seasons, while predators at higher trophic levels were more “integrators” with added metabolic complexity leading to somewhat more divergent FA profiles. These results are potentially applicable for other aquatic consumers in freshwater and marine ecosystems.

Aquatic macroinvertebrates play an important role in trophic transfer within and across ecosystems, linking energy flow from basal food sources to upper trophic levels such as fish and eventually humans (Allan and Castillo 2007), and providing carbon and essential nutrients to terrestrial insects and birds (Baxter et al. 2005; Twining et al. 2016). However, few empirical studies have evaluated the broad‐scale conditions that enhance or limit coupling between macroinvertebrate nutritional quality and their food sources. Although macroinvertebrates adapt to seasonally available food pulses in many environments, these pulses are often difficult to estimate and their pathway to higher trophic levels is still poorly known (Ostfeld and Keesing 2000). This is particularly true in stream ecosystems where the availability and quality of food sources for macroinvertebrates often changes seasonally because of changes in the physical and chemical conditions within the watershed (Dodds and Whiles 2010). Furthermore, compared with lentic ecosystems, streams, which have greater perimeter to area ratios are more directly connected to their catchments (Dodds and Whiles 2010). For instance, removing riparian trees causes major changes in light availability in streams, but generally less so in lakes or wetlands. Also degradation of stream riparian zones can lead to an increase of terrestrially derived nutrients and pollutants, which consequently affect basal food sources for macroinvertebrates (Zhao et al. 2017). Such impacts to basal food sources can affect the nutritional quality of macroinvertebrates and have important consequences for higher trophic levels.

Assessment of resource long‐chain polyunsaturated fatty acid (LC‐PUFA) content is an increasingly important and informative approach to assess dietary quality for animals (Brett et al. 2017). High‐dietary LC‐PUFA content indicates high‐food quality for consumers and enhances somatic growth (Müller‐Navarra et al. 2000; Brett et al. 2009). LC‐PUFA, in particular EPA (20 : 5ω3), docosahexaenoic acid (DHA, 22 : 6ω3), and arachidonic acid (ARA, 20 : 4ω6) are generally essential for invertebrate development, reproduction, and hormone regulation, especially for insect emergence and reproduction (Stanley‐Samuelson 1994). However, many aquatic animals only have a limited ability to synthesize LC‐PUFA from the shorter chain PUFA alpha‐linolenic acid (ALA, 18 : 3ω3) and linoleic acid (LIN, 18 : 2ω6) (Brett and Müller‐Navarra 1997; Wacker et al. 2002; Torres‐Ruiz et al. 2010), and they must therefore obtain most of their LC‐PUFA directly from their diet. High LC‐PUFA retention in metazoan consumers means high availability for the next trophic level (Brett and Müller‐Navarra 1997; Taipale et al. 2011). However, even though LC‐PUFA are highly retained in macroinvertebrates (Guo et al. 2017), little is known how macroinvertebrates utilize dietary LC‐PUFA. Do macroinvertebrates simply collect LC‐PUFA from their food sources (“LC‐PUFA collector”), or are they able to regulate their PUFA content relative to food sources (“LC‐PUFA integrator”)? The term “LC‐PUFA collector” would signify matching diet and consumer LC‐PUFA profiles, thus describing a consumer that has a limited ability to modify dietary LC‐PUFA. In contrast, we define the term “LC‐PUFA integrator” as a consumer that is able to selectively retain dietary LC‐PUFA or maintain a quasi‐homoeostatic LC‐PUFA for its physiological requirements. This latter strategy could thus result in a mismatch between diet and consumer LC‐PUFA profiles.

Macroinvertebrates are selective feeders and may preferentially feed on high‐quality food for LC‐PUFA (Bunn et al. 2013; Guo et al. 2016 *c*; Brett et al. 2017). Periphyton and terrestrial organic matter are two primary basal food sources in stream ecosystems. Recent studies have suggested that periphyton is the main carbon source for macroinvertebrates in many streams and rivers with terrestrial organic matter playing only a minor role (Bunn et al. 2003; Delong and Thorp 2006; Lau et al. 2009 *a*). Compared with terrestrial carbon in the form of leaf litter/plant materials, periphyton is a high‐quality food source for macroinvertebrates because of its much higher LC‐PUFA content, especially EPA (Torres‐Ruiz et al. 2007; Lau et al. 2009 *a*; Guo et al. 2016 *b*). In stream periphyton and invertebrates, DHA is rather low or absent, and its functional role for invertebrate neural development may be fulfilled by EPA (Stanley‐Samuelson 1994; Ahlgren et al. 2009). EPA is almost exclusively synthesized by certain taxa of algae, particularly diatoms and cryptophytes (Taipale et al. 2013). In contrast, fresh terrestrial leaves of vascular plants are typically characterized by nondetectable levels of EPA and DHA, and relatively high levels of ALA and LIN (Napolitano 1999). Submerged or conditioned leaves can increase in nutritional quality in terms of lower C : N ratios and increased protein content, following fungi and bacteria colonization (Manning et al. 2015; Tant et al. 2015). However, fungi and bacteria are still usually poor quality diets for invertebrates in terms of fatty acids (FAs) since they generally lack LC‐PUFA (Cooney et al. 1993; Desvilettes et al. 1997; Kainz and Mazumder 2005). Therefore, macroinvertebrates may selectively feed on high‐quality periphyton for their LC‐PUFA requirement.

The LC‐PUFA content of periphyton is sensitive to seasonal and spatial variation in stream environmental conditions (Hill et al. 2011; Cashman et al. 2013; Guo et al. 2015). For example, the extent of FA unsaturation in algae can vary with temperature. Low temperatures result in an increase in algal EPA content, but decreases in ALA and LIN (Piepho et al. 2012). Moreover, spatial differences in riparian canopy cover along the river continuum may regulate periphyton LC‐PUFA synthesis. High‐canopy cover generally increases the relative content of EPA and ARA and reduces ALA and LIN (Hill et al. 2011; Guo et al. 2015), whereas low canopy is required for the synthesis of saturated fatty acids (SAFAs), which are often used for energy storage (Brett and Müller‐Navarra 1997). In addition, periphyton LC‐PUFA content is also influenced by nutrient levels, pollutant inputs, and streambed drying (Guschina and Harwood 2006; Sanpera‐Calbet et al. 2017). Despite these apparent broad patterns, it is still largely unknown how changes in periphyton FA would consequently affect the FA composition of macroinvertebrates. Although several studies have found that macroinvertebrate FA profiles match their dietary FA (Torres‐Ruiz et al. 2007; Torres‐Ruiz et al. 2010; Guo et al. 2016 *a*), the resolution of periphyton FA on macroinvertebrate FA along spatial and temporal changes of their habitats is poorly understood.

Current field studies, mostly focusing on the dynamics of macroinvertebrate FA, suggest that in addition to periphyton, sampling season and location, and particularly phylogenetic differences also affect macroinvertebrate FA composition (Sushchik et al. 2006; Sushchik et al. 2007; Torres‐Ruiz et al. 2007; Makhutova et al. 2011; Smits et al. 2015; Twining et al. 2017). For instance, it was noted that the FA composition differed significantly between Trichoptera and Ephemeroptera (Smits et al. 2015). However, most current studies primarily highlight the FA dissimilarity at species levels, without recognizing trophic relationships and feeding strategies. Although some macroinvertebrates may differ in FA metabolism abilities at the species level (Makhutova et al. 2011), their functional role in linking the energy flow from basal food sources to higher trophic levels should be acknowledged when interpreting macroinvertebrate FA data. Important factors that may affect macroinvertebrate FA profiles, such as periphyton assimilation, species identity, functional feeding groups (FFGs), and seasonal and spatial environmental variations, should be thus considered in food web studies. To our knowledge, the relative importance of these factors has not been characterized simultaneously, which is key to understand which factors are most important.

To address this, we conducted a field study in subalpine streams in Austria to examine how the nutritional quality (measured by LC‐PUFA) of macroinvertebrates changes relative to their basal food sources. The key objectives of this study were to (1) determine the FA dissimilarities between macroinvertebrates and their basal food sources, i.e., periphyton and terrestrial leaves, (2) quantify the influence of periphyton FA on macroinvertebrate FA, and (3) determine the relative influence of periphyton FA on macroinvertebrate FA composition compared with other factors, i.e., invertebrate species identity, and seasonal and spatial environmental variation.

## Methods

### Study streams

This study was conducted in the subalpine River Ybbs catchment, Austria (47°45′N, 15°12′E), with a drainage area of 254 km^2^. The climate is temperate, with distinct seasonal changes, though precipitation is typically evenly distributed over the year. Catchment geology is dominated by dolomite and karst. Forestry is the major land use in the upper catchment, while alpine meadows and agricultural areas constitute only a small fraction of the catchment (Besemer et al. 2013). Our stream sites were selected in the upper catchment, *Weiße Ois*, with only minor human disturbance in the study reaches or their upstream catchments.

### Sample collection

Macroinvertebrates, periphyton, and leaves were collected from 17 riffles in the catchment in July and October 2016 (Table 1). Three quadrats (1.5 m × 1.5 m) were sampled within a 20‐m reach at each riffle. Rocks in the quadrat were randomly picked, and macroinvertebrates clinging to the rocks were washed into a white tray. Mayflies, caddisflies, and stoneflies were identified to genus, and other macroinvertebrates were sorted to order. Macroinvertebrates of different genera or orders from different quadrats were put into individual vials as individual samples for FA analyses. A separate invertebrate sample was preserved for further taxonomic identification. The same rocks were then used to collect periphyton for FA analyses. Three replicated samples were collected from the three quadrats, respectively, and each sample was collected from five different cobbles. Periphyton was scraped with brushes from the cobbles. All samples were immediately placed in zip‐lock plastic bags, stored on ice, and kept in the dark in a portable freezer. Samples were brought to the laboratory within 4 h. All FA samples were placed in a − 80°C freezer until further processing, and the separate invertebrate sample was preserved in 75% ethanol.

**Table 1:** Field‐measured environmental characteristics and ambient nutrient concentrations obtained from 17 study streams over two seasons in 2016 in the Ybbs catchment, Austria.

Streams	Latitude	Longitude	Canopy	Temperature (°C)		Velocity (m/s)		pH		DIN (mg/L)		SRP (μg/L)	
				Summer	Fall	Summer	Fall	Summer	Fall	Summer	Fall	Summer	Fall
Bodingbach	47.8618	15.0209	High	19.80	7.70	0.81	0.74	8.52	8.52	1.13	1.10	3.70	2.93
Faltlbach	47.7838	15.1813	Low	14.40	7.90	0.19	0.36	8.38	8.43	0.89	0.72	1.00	0.00
Ois Holzhüttenboden	47.8113	15.1481	Middle	15.50	4.30	0.35	0.37	8.79	7.20	0.69	0.37	1.40	2.23
Kothbergbach oben	47.8636	14.9605	High	11.20	6.50	0.20	0.29	8.42	8.39	1.05	0.82	0.43	2.07
Kothbergbach unten	47.8778	14.9946	High	12.90	6.70	0.53	0.63	8.70	8.70	0.69	1.12	1.40	0.73
Lackenbach	47.8594	15.1105	High	10.10	8.20	0.71	0.83	8.40	8.43	0.93	0.83	1.47	9.30
Alte Säge	47.8590	15.1103	Low	10.10	8.20	0.45	0.62	8.05	8.14	0.95	0.74	‐	1.90
Oberer Seebach Lend	47.8511	15.0706	Middle	10.00	7.50	0.48	0.71	8.16	8.21	1.08	0.84	1.53	1.90
Oberer Seebach Ritrodat	47.8524	15.0655	Middle	12.20	7.40	0.26	0.25	8.31	8.31	0.93	0.81	1.60	1.83
Weiße Ois Rehberghütte	47.7684	15.1574	Low	11.80	6.40	0.22	0.33	8.87	8.42	0.73	0.67	0.00	0.00
Tagles Schelchen	47.8196	15.1122	Middle	11.50	5.90	0.28	0.35	8.13	8.50	0.92	0.54	0.70	0.67
Taschlbach	47.7892	15.1915	Middle	12.50	4.50	0.33	0.35	8.44	7.18	0.82	0.27	3.17	1.43
Tagles unten	47.8319	15.1245	Low	14.00	4.70	0.29	0.33	8.38	7.20	0.62	0.35	1.13	0.87
Weiße Ois unterhalb Faltl	47.7657	15.1785	Low	11.80	7.20	0.30	0.44	8.09	8.19	0.78	0.65	0.87	0.00
Ybbs Göstling ‐ Lagerhaus	47.8079	14.9370	Low	11.50	6.90	1.82	0.65	8.37	8.53	0.86	0.81	0.77	1.07
Ybbs Lunz ‐ Großau	47.8292	15.0022	Low	13.10	6.30	0.73	0.62	8.37	8.55	0.87	0.82	0.93	1.30
Ybbs Lunz ‐ Kläranlage	47.8550	15.0209	Low	11.20	7.10	0.65	0.52	8.37	8.37	0.93	0.92	1.43	1.87

DIN, dissolved inorganic nitrogen; SRP, soluble reactive phosphorus.

In July, fresh leaves were collected from riparian trees and grasses, since very few submerged leaves were observed in the streams. In late October, submerged leaves were collected from each stream by hand. All leaf samples were sorted, kept in the dark in a portable freezer in the field, and stored at − 80°C in a freezer in the laboratory. Leaf samples included leaves from alder (*Alnus alnobetula*), spruce (*Picea abies*), willow (*Salix caprea*), maple (*Acer pseudoplatanus*), hazel (*Corylus avellana*), ivy (*Hedera helix*), beech (*Fagus sylvatica*), butterbur (*Petasites hybridus*), mountain pine (*Pinus mugo*), and reeds (*Phragmites australis*).

At each stream, triplicate water samples were collected to determine the concentrations of dissolved inorganic nutrients, specifically nitrate (NO_3_–N), nitrite (NO_2_–N), ammonium nitrogen (NH_4_–N), and soluble reactive phosphorus (SRP). Samples were stored in a portable dark cooler in the field and filtered through 0.7‐*μ*m glass microfiber filters (GFF; Whatman^TM^, GE Healthcare) in the laboratory. Samples were analyzed within 2 d of collection.

Riparian canopy cover was estimated in situ (Table 1). Sites were classified as “high canopy” when their two riverbanks were surrounded by high trees, whereas they were coded as “middle canopy” if only one riverbank of the site was covered by high trees. Sites without surrounding trees on both riverbanks were recorded as “low canopy.” Temperature and pH were measured using a HQD portable measuring meter (HQ30D—Multi/1 Channel, HACH LANGE, Germany) (Table 1). Current velocity was measured by a mechanical flow meter (Hydro‐Bios, Kiel, Germany) (Table 1).

### Sample processing

Macroinvertebrates in the separate sample were identified to genus using a dissecting microscope (Leica MZ6; Leica Microsystems, Wetzlar, Germany), and the results were used to validate the invertebrate samples used for FA analyses. Macroinvertebrates were assigned to FFG as determined by Cummins and Klug (1979). Invertebrate grazers found in our study streams included *Baetis* sp. and *Ecdyonurus* sp. and shredders included *Leuctra* sp., *Nemoura* sp., *Potamophylax* sp., and *Allogamus* sp. Predators included *Perla* sp., *Plectrocnemia* sp., *Perlodes* sp., *Rhyacophila* sp., and *Isoperla* sp. The only filterer collected was *Hydropsyche* sp. The macroinvertebrates that were present in more than 70% of sites in the two seasons were *Baetis* sp., *Ecdyonurus* sp., *Leuctra* sp., *Perla* sp., *Rhyacophila* sp., and *Plectrocnemia* sp.

All FA samples for macroinvertebrates, periphyton, and leaves were freeze‐dried (Virtis Genesis Freeze Dryer). After freeze‐drying, invertebrate taxonomy was further checked and then each sample was homogenized with a glass rod. Leaf samples were ground to powder and homogenized with a food processor. From each invertebrate sample 5–7 mg of dry mass, 10 mg dry mass from each periphyton sample, and 50 mg dry mass from each leaf sample were used for lipid extraction, respectively. Lipids were extracted and methylated according to the methods reported in Guo et al. (2016 *c*). Fatty acid methyl esters (FAME) were analyzed using a gas chromatograph (THERMO Trace; FID 260°C, carrier gas: He: 1 mL/min, detector gases: H_2_: 40 mL/min, N_2_: 45 mL/min, air: 450 mL/min, temperature ramp: 140°C (5 min)–4°C/min–240°C (20 min) = 50 min) equipped with a temperature‐programmable injector and an autosampler. FAME were separated by a Supelco^TM^ SP‐2560 column (100 m, 25 mm i.d., 0.2 μm film thickness), identified by comparison of their retention times with known standards (37‐component FAME mix, Supelco 47885‐U; Bacterial Acid Methyl Ester Mix, Supelco 47080‐U) and quantified with reference to seven‐point calibration curves based on known standard concentrations. FA compositions were expressed as percentages relative to total FA (FA%).

Dissolved nutrients (NO_3_–N, NO_2_–N, NH_4_–N, and SRP) were analyzed using a continuous flow analyzer (Alliance instrument GmbH, 5020, Salzburg, Austria) (APHA 1995; Table 1).

### Data analysis

All FA percentage data were arcsine‐square‐root‐transformed for normal distribution approximation before analysis (Kelly and Scheibling 2012; Lau et al. 2012). Eight FA groups were used in analyses, i.e., LIN, ALA, ARA, EPA, DHA, SAFA, sum of monounsaturated FA (MUFA), and sum of bacterial FA (BAFA). BAFAs included 15:0, 17:0 and their branched *iso*‐ and *anteiso*‐homologues, and 18 : 1ω7. These FA groups represented essential FA and important FA functional groups in macroinvertebrates and basal food sources (Guo et al. 2016 *b*).

Differences in FA compositions of aquatic macroinvertebrates, periphyton, and terrestrial leaves were first detected by principal component analysis (PCA). Significance of the resulting eigenvalues was tested using Monte Carlo randomization with 1000 permutations. Furthermore, analysis of similarity (ANOSIM) was applied to examine the similarities in FA compositions between macroinvertebrates and basal food sources. ANOSIM calculates an R statistic that assesses the differences between groups, where R‐values close to 1 indicate complete dissimilarity among sample sets and values near 0 suggest no difference among sample sets (Clarke and Warwick 2001). Additionally, linear mixed‐effect models were further used to examine FA differences in invertebrate FFG and their basal food sources across sites and seasons. In our study, the response variables were individual FA% (e.g., EPA, ARA, DHA, ALA, and LIN, respectively), with consumers and dietary resources (invertebrate FFG, periphyton, fresh leaves, and submerged leaves) as the fixed factors and sites and seasons as the random factors. The protocol for linear mixed‐effect model fit and validation followed Zuur et al. (2009) and Guo et al. (2016 *a*). Restricted maximum likelihood estimation was used to fit models, and the likelihood ratio test was used to compare and select fitted models. Post‐hoc multicomparison among taxa was conducted when the fixed factor showed a significant effect on the response variable (e.g., EPA).

The effect of seasonal and spatial environmental changes on periphyton FA composition was assessed by redundancy analysis (RDA). Preliminary detrended correspondence analysis of periphyton FA data set produced the gradient lengths of 0.608 and 0.436 for the first two axes, respectively, suggesting that the response was monotonic and thus a linear model (rather than an unimodal model) was more suitable to explain the FA data structure (Ter Braak and Prentice 1988; Guo et al. 2015). Therefore, a multivariate direct gradient analysis, RDA (Legendre and Legendre 1998), was used to analyze the data set. In RDA, periphyton FA were used as response variables, with environmental factors as independent variables. The significance of the RDA model and canonical axes were tested using permutation procedures, with the marginal effects of all measured environmental factors tested in the same manner.

The effect of periphyton FA variation on invertebrate FA was evaluated by piecewise structural equation modeling (SEM). This approach constructs the path model as a set of hierarchical linear mixed models, each of which was fitted using restricted maximum likelihood, using the nlme package (version 3.1–117) (Pinheiro et al. 2014) in R, and the overall path model (the SEM) was fitted using the R package piecewise SEM (Lefcheck 2016). Specifically, an a priori directed acyclic graph (Shipley 2000) was first constructed according to previous studies that have shown the trophic links of LC‐PUFA from basal food sources (periphyton and leaves) to invertebrate primary consumers (grazers, shredders, and filterers) and then to secondary consumers (predators) (Torres‐Ruiz et al. 2007; Lau et al. 2009 *b*). Then the graph was translated into a set of linear models. As samples from adjacent sites are likely to share similar characteristics, our piecewise SEM was fitted using linear mixed‐effect models, with site as the random factor for each model. Model fit was evaluated by the test of directed separation, based on the Fisher's C statistic and a chi‐square distribution significance test (Shipley 2009). The procedure tests the assumption that all variables are conditionally independent. In simplest terms, conditional independence implies that there are no missing relationships among unconnected variables (Shipley 2000). When there is weak support for the set of the conditional independence claims, the *p*‐value for the chi‐square test is greater than the chosen significance threshold (typically *p* = 0.05) (Shipley 2000, 2009). Path coefficients were then calculated for each model.

The correlations between dietary ALA and primary consumer EPA were calculated to (1) assess if macroinvertebrates were able to convert EPA from the short‐chain precursor ALA, and (2) validate the results from piecewise SEM. If consumers were capable of converting EPA from dietary ALA, significant correlations would be observed, and the piecewise SEM would be reconstructed.

In addition, the effect of temperature and riparian canopy on invertebrate FA was estimated by linear mixed‐effect models, respectively, with temperature/riparian canopy as the fixed factor and site as the random factor. Post‐hoc multicomparison was applied to test the FA differences among temperature or riparian canopy.

The relative importance of periphyton FA, seasonal and spatial environmental factors, and invertebrate species identity for macroinvertebrate FA composition was partitioned and quantified by partial redundancy analysis (partial RDA). The invertebrate FA data were used as the response variable, whereas the four sets of data, i.e., periphyton FA, seasonal and spatial environmental factors, and invertebrate species identity, were explanatory variables. Temperature was the seasonal factor and spatial environmental factors included riparian canopy, DIN, SRP, velocity, and pH. Species identities were coded as dummy environmental variables (Jongman et al. 1995), which included 13 taxa. Partial RDA was performed based on a series of combinations of different explanatory variables with or without covariables to partition the influence of explanatory variables.

All statistical analyses were conducted in the statistical software R version 3.3.3 (R Core Team 2017), using the extension package vegan for PCA, RDA and partial RDA (Oksanen et al. 2013), lme4 and nlme for linear mixed‐effect models (Bates et al. 2012; Pinheiro et al. 2014), multcomp for post‐hoc multicomparison (Hothorn et al. 2008), and piecewise SEM for piecewise SEM (Lefcheck 2016).

## Results

### Differences in FA compositions among macroinvertebrates and basal food sources

**Figure 1:**
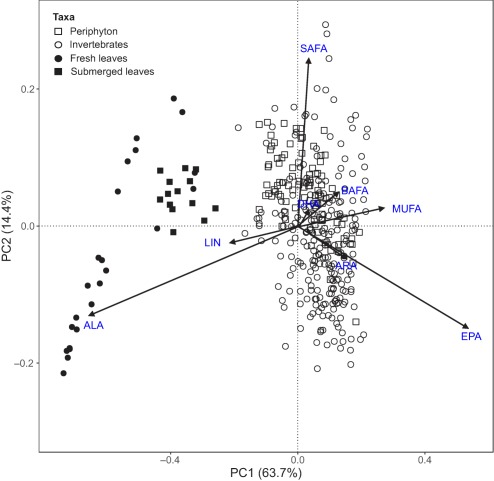
PCA on all FA samples of invertebrates, periphyton, fresh leaves, and submerged leaves from 17 study streams over two seasons in 2016 in the Ybbs catchment, Austria.

Systematic differences between terrestrial and aquatic FA were observed in the PCA (Fig. 1). Only the first two eigenvalues were significant (PC1, *p* < 0.001; PC2, *p* < 0.001), and the first two PCs explained 63.7% and 14.4% of the total variance, respectively. PC1 was positively associated with EPA (correlation coefficient: 0.89), and negatively correlated with ALA (− 0.91) and LIN (− 0.62), which separated terrestrial leaves from aquatic periphyton and macroinvertebrates. Periphyton and macroinvertebrates contained considerably higher EPA% (Table 2), whereas EPA% in fresh leaves was absent and in submerged leaves was < 1%, but both leaves had much higher ALA% and LIN% than aquatic organisms. PC2 was positively correlated with SAFA (0.86), indicating the FA differences between periphyton and macroinvertebrates. Further, ANOSIM showed that invertebrate FA were significantly dissimilar to the FA composition of fresh (0.997, *p* = 0.001) or submerged leaves (0.973, *p* = 0.001), but more similar to periphyton FA (0.172, *p* = 0.001). Likewise, periphyton FA were significantly dissimilar to fresh (0.998, *p* = 0.001) or submerged leaves (0.981, *p* = 0.001). The FA composition of fresh and submerged leaves was also dissimilar (0.652, *p* = 0.001).

**Table 2:** FA composition (% relative to total FAs, mean ± SE) of macroinvertebrates, periphyton, and leaf samples from 17 study streams over two seasons in 2016 in the Ybbs catchment, Austria. Linear mixed‐effect models were applied to examine differences in individual FA composition of invertebrates and basal food sources. Different superscript letters indicate significant differences among groups (*p* < 0.05). Fresh leaves were collected in July, and submerged leaves were collected in October.

FA%	Fresh leaves (*n* = 23)	Submerged leaves (*n* = 14)	Periphyton (*n* = 100)	Grazers (*n* = 66)	Filterers (*n* = 23)	Shredders (*n* = 51)	Predators (*n* = 93)
LIN	16.05 ± 6.60^a^	10.50 ± 1.20^b^	7.56 ± 2.47^c^	3.51 ± 0.92^e^	5.39 ± 2.32^d^	9.53 ± 4.49^b^	6.66 ± 2.45^c,d^
ALA	42.82 ± 15.41^a^	31.01 ± 3.44^b^	9.63 ± 3.11^c^	10.91 ± 4.47^c^	8.69 ± 3.43^c^	11.18 ± 4.29^c^	11.9 ± 3.06^c^
ARA	0.04 ± 0.12 ^a^	0.23 ± 0.22^b^	0.73 ± 0.50^c^	0.72 ± 0.45^c^	0.70 ± 0.51^c^	1.27 ± 0.98^d^	1.28 ± 0.81^d^
EPA	0.00^a^	0.61 ± 0.65^a^	10.29 ± 4.27^b^	14.09 ± 4.16^c^	9.96 ± 5.35^b^	8.98 ± 4.79^b^	17.25 ± 4.46^d^
DHA	0.00^a^	0.04 ± 0.08^a,d^	0.50 ± 0.25^b^	0.04 ± 0.03^d^	0.12 ± 0.20^c^	0.12 ± 0.11^c^	0.13 ± 0.19^c^
SAFA	29.85 ± 8.81^a,e^	33.69 ± 2.23^a,b,d^	36.06 ± 4.93^b^	35.17 ± 5.91^b,c^	40.56 ± 7.27^c^	31.37 ± 6.93^e^	29.14 ± 5.31^d,e^
MUFA	8.51 ± 6.23^a^	13.03 ± 1.91^b^	26.27 ± 3.20^c^	30.45 ± 4.39^d^	29.27 ± 4.49^c,d^	31.21 ± 5.33^e^	29.06 ± 4.30^d,e^
BAFA	1.28 ± 0.75^a^	11.66 ± 1.04^b^	8.05 ± 2.98^c^	12.07 ± 1.38^b^	7.60 ± 1.72^c,e^	6.84 ± 3.07^e^	7.04 ± 1.95^e^

ALA, alpha‐linolenic acid; ARA, arachidonic acid; BAFA, bacterial FA; DHA, docosahexaenoic acid; EPA, eicosapentaenoic acid; LIN, linoleic acid; MUFA, monounsaturated FA; SAFA, saturated fatty acid.

Results of linear mixed‐effect models were consistent with the results of PCA and ANOSIM, and further indicated individual FA differences amongst invertebrate FFG and periphyton (Table 2). Invertebrate predators had higher proportions of EPA, ALA and ARA compared with periphyton, and other FFG. Grazers were characterized by relatively high EPA% and BAFA%, but low LIN%, whereas shredders contained high proportions of LIN and ARA, but low SAFA%. As for filterers, their EPA, ALA, and ARA contents were similar to periphyton and intermediate compared to other FFG.

### Effects of seasonal and spatial environmental changes on periphyton FA and their consequent effect on macroinvertebrate FA

**Figure 2:**
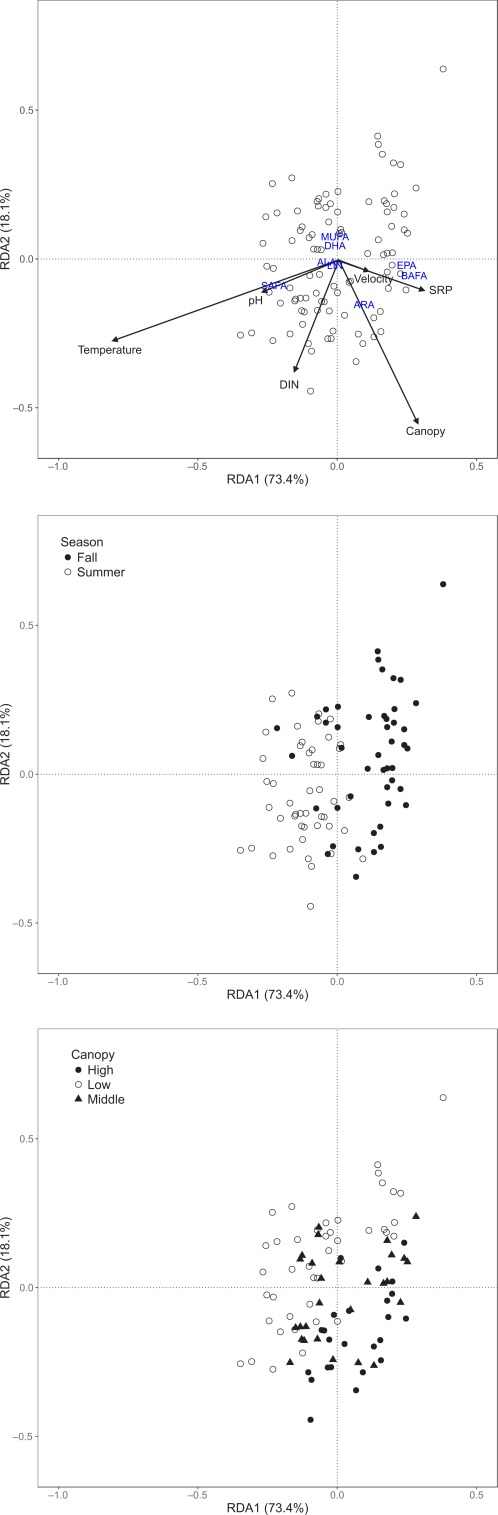
Ordination plots of canonical axes 2 vs. 1 from the RDA of periphyton FA composition for all samples from 17 study streams over two seasons in 2016 in the Ybbs catchment, Austria. (**A**) Ordination plots with FA responses; (**B**) Position of study streams in each season on the first two axes (RDA1 and RDA2); (**C**) Position of study streams relative to riparian canopy on the first two axes (RDA1 and RDA2).

An RDA of the FA composition of all periphyton samples indicated that temperature and riparian canopy were the most important environmental predictors of periphyton FA profiles (Fig. 2). Only the first two axes were significant (RDA1: *F*‐value = 21.009, *p* = 0.001; RDA2: *F*‐value = 5.171, *p* = 0.003) with the explained FA variation 73.4% and 18.1%, respectively. The marginal effects for all environmental variables showed that only temperature (*F*‐value = 12.707, *p* = 0.001) and riparian canopy (*F*‐value = 5.714, *p* = 0.003) were significant. RDA1 was strongly correlated with temperature (− 0.796) with the second axis mainly correlated with riparian canopy (− 0.876) (Fig. 2A). RDA1 separated periphyton FA from summer to fall (Fig. 2B). Periphyton EPA% and BAFA% tended to increase from summer to fall. RDA2 reflected the FA differences among different riparian canopy types (Fig. 2C). Periphyton ARA% increased as riparian canopy increased from low to middle and high canopy within seasons.

**Figure 3:**
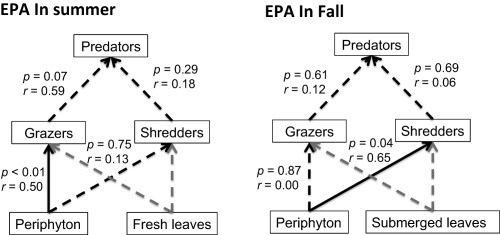
EPA (20 : 5ω3) pathways from basal food sources to invertebrate consumers. Solid paths are statistically different from 0 at *p* < 0.05, whereas dashed paths are not. Gray dashed paths indicate those paths do not exist.

Piecewise SEM was established for EPA in summer and fall, respectively, as temperature had a significant seasonal effect on periphyton FA. Filterers were not included in the analysis given their small sample size. EPA was chosen for piecewise SEM for three reasons: (1) no significant correlations between periphyton ALA and primary consumer EPA were observed (for grazers, the correlation coefficient − 0.10, *p* = 0.56; shredders, − 0.34, *p* = 0.07; and filterers, 0.11, *p* = 0.60). Similarly, no significant correlations between leaf ALA (fresh and submerged leaves, respectively) and primary consumer EPA were found (fresh leaves: for grazers, − 0.36, *p* = 0.19; shredders, − 0.27, *p* = 0.35; and filterers, 0.53, *p* = 0.14. Submerged leaves: for grazers, − 0.28, *p* = 0.36; shredders, − 0.37, *p* = 0.29; and filterers, − 0.39, *p* = 0.24). These results suggested that macroinvertebrate primary consumers were not very efficient at converting EPA from dietary ALA, and they must therefore directly obtain EPA from diet sources; (2) results of PCA and ANOSIM showed that macroinvertebrate FA was dissimilar to leaf FA but more similar to periphyton FA; EPA% was considerably high in periphyton and macroinvertebrates, and very low in submerged leaves and absent in fresh leaves; (3) compared with other FA, EPA is critical for the somatic growth and reproduction of stream macroinvertebrates (Stanley‐Samuelson 1994; Guo et al. 2016 *b*). Piecewise SEM yielded final path models for EPA, which were well supported by our data (Fig. 3). Results of directed separation tests showed that Fish's C = 3.23 and *p* = 0.52, which indicated the final causal models were significant. The direct impact of periphyton on macroinvertebrate predators was not significant, but this effect indirectly occurred through primary consumers. Grazer EPA was significantly affected by periphyton in summer (*r* = 0.50, *p* < 0.01), whereas shredder EPA was significantly related to periphyton in fall (*r* = 0.65, *p* = 0.04). However, grazers and shredders did not show any effects on predator FA.

**Figure 4:**
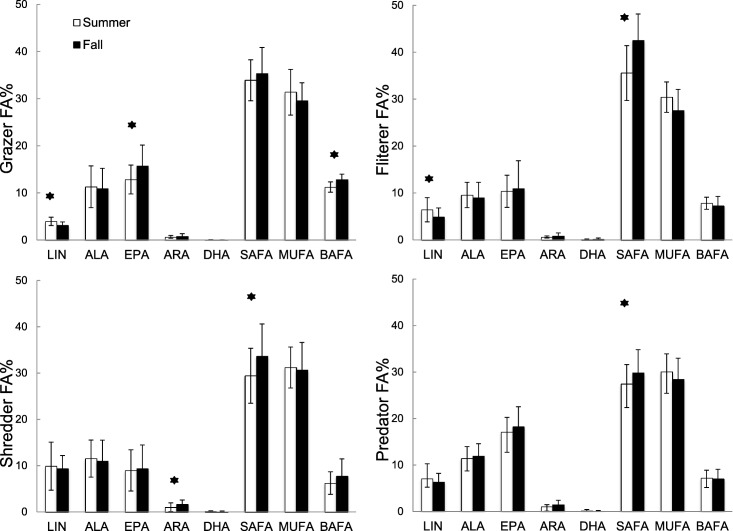
Seasonal changes in invertebrate FA composition from 17 study streams over two seasons in 2016 in the Ybbs catchment, Austria. Linear mixed‐effect models were applied to examine differences in individual FA percentage composition of invertebrates from summer to fall.

Results of linear mixed‐effect models showed invertebrate FFG differed in their response to temperature change (Fig. 4) but did not show any significant responses to riparian canopy. Consistent with periphyton FA, grazer EPA% and BAFA% significantly increased (0.03, *p* < 0.001; 0.02, *p* < 0.001) from summer to fall, whereas LIN% decreased (− 0.02, *p* < 0.001). SAFA% in all FFG except grazers increased significantly from summer to fall (grazers: 0.02, *p* = 0.11; shredders: 0.04, *p* = 0.04; filterers: 0.06, *p* = 0.02; predators: 0.02, *p* = 0.02).

### Partitioning effects of periphyton FA, seasonal and spatial environmental factors, species identity on invertebrate FA composition

The summed periphyton FA, spatial and seasonal factors, and invertebrate species identity together explained 68.9%, 57.9%, and 39.5% of FA variations in grazers, shredders, and predators, respectively (Table 3). The joint effects of periphyton FA and invertebrate species identity accounted for most of the total effects of four explanatory variables, and contributed 62.3% for grazer FA variations, 46.0% for shredders, and 33.7% for predators. Specifically, periphyton FA alone contributed 38.5% of the explained FA variations in grazers, which was 1.9 times higher than the periphyton FA effect on shredders (20.5%), and 2.2 times higher than that on predators (17.5%). In contrast, invertebrate species identity alone contributed 26.2% and 27.1% of FA variations in grazers and shredders, respectively, which was higher than the effect on predators (17.9%). In addition, the joint effects of seasonal and spatial environmental factors explained 19.6%, 13.4%, and 11.0% of FA variations in grazers, shredders, and predators, respectively. Season alone showed a low effect on invertebrate FA composition.

**Table 3:** Partial RDA results of the contributions of periphyton FAs, seasonal and spatial environmental factors, and invertebrate species identity to the FA compositions of macroinvertebrates collected from 17 study streams over two seasons in 2016 in the Ybbs catchment, Austria. Temperature was the seasonal factor, and spatial environmental factors included riparian canopy, DIN, SRP, velocity, and pH.

	Grazers	Shredders	Predators
*Total effect (%)*	68.86	57.94	39.47
*Periphyton FA*	38.50	20.49	17.48
Periphyton FA + invertebrate species identity	62.25	45.98	33.70
Periphyton FA + spatial factors	43.09	31.79	22.46
Periphyton FA + seasons	39.80	23.32	18.36
Periphyton FA + invertebrate species identity + spatial factors	67.20	56.81	38.68
Periphyton FA + invertebrate species identity + seasons	63.53	48.68	34.57
Periphyton FA + spatial factors + seasons	44.72	32.45	23.36
*Invertebrate species identity*	26.18	27.14	17.94
Invertebrate species identity + spatial factors	41.96	38.73	25.23
Invertebrate species identity + seasons	30.45	31.44	20.29
Invertebrate species identity + spatial factors + seasons	46.34	41.33	28.41
*Spatial environmental factors*	15.21	11.54	7.83
Spatial factors + seasons	19.62	13.40	10.96
*Seasons*	4.32	2.42	2.39

FA, fatty acid.

## Discussion

Our study suggests that stream macroinvertebrates exhibit a mixed feeding strategy, rather than simply collecting or integrating high‐quality LC‐PUFA, especially EPA, relative to their basal food sources. Although the periphyton FA effect on macroinvertebrate FA profiles decreased with increasing trophic levels, the EPA% in macroinvertebrate body tissues increased (Gladyshev et al. 2011; Lau et al. 2012; Brett et al. 2017; Guo et al. 2017), indicating EPA accumulation is a general ecological response in stream food webs. We propose three hypotheses regarding the mechanism that macroinvertebrates use to accumulate EPA: (1) macroinvertebrates may selectively feed on high‐quality periphyton instead of terrestrial leaves, (2) although macroinvertebrates feed on a mixed periphyton assemblage, they may assimilate EPA more efficiently than other FA, or preferentially assimilate EPA over other FA, and/or, (3) macroinvertebrates may actively control their LC‐PUFA distribution through metabolic processing to support their somatic growth, reproduction, and survival. The relative importance of those three possibilities may differ among FFG across seasons.

Differences in the FA composition among aquatic macroinvertebrates, periphyton, and terrestrial leaves suggest that periphyton is the main LC‐PUFA (in particular EPA) source for macroinvertebrates. Although submerged leaves were not available in our study streams in summer and FA of biofilms attached to submerged leaves were not analyzed, previous studies report that submerged leaves as a whole contained no or very little EPA, < 1% (Brett et al. 2009; Torres‐Ruiz and Wehr 2010; Guo et al. 2016 *c*), which is also confirmed by our leaf FA (Table 2). Moreover, seasonal change is unlikely to affect the low‐EPA content of submerged leaves (Torres‐Ruiz and Wehr 2010). Macroinvertebrate primary consumers in our study streams showed limited ability to synthesize EPA from dietary ALA, suggesting they obtained EPA from food sources that are rich in EPA (Brett and Müller‐Navarra 1997; Wacker et al. 2002; Torres‐Ruiz et al. 2010). Terrestrial leaves may serve as shelters or play other roles for macroinvertebrates, but offer only weak dietary contributions to macroinvertebrate EPA (Torres‐Ruiz et al. 2007; Lau et al. 2008; Brett et al. 2017). Our finding complements previous isotope studies that demonstrate periphyton is the primary carbon source for macroinvertebrates in many streams (Bunn et al. 2003; Delong and Thorp 2006; Lau et al. 2009 *a*). Given the lack of LC‐PUFA in terrestrial leaves, periphyton indirectly and stream macroinvertebrates directly may also provide a significant LC‐PUFA subsidy to terrestrial animals in riparian habitats (Baxter et al. 2005; Twining et al. 2016).

In our study streams, seasonal temperature change explained most of the variation in periphyton FA, followed by riparian canopy. Our results provide field evidence that periphyton in fall (low temperature) contained more EPA% compared with periphyton in summer (high temperature), since most previous studies focused primarily on individual algal species in the laboratory (Guschina and Harwood 2006; Piepho et al. 2012). As for riparian canopy, periphyton ARA% increased as riparian canopy increased within seasons, which is consistent with earlier studies in temperate and subtropical streams (Hill et al. 2011; Guo et al. 2015). Nutrient concentrations did not show significant effects on periphyton FA, probably because of the very low human perturbation in our study area.

Our results suggest that the periphyton FA effect on the FA composition of macroinvertebrate grazers may lead to different feeding strategies for EPA requirements across seasons, and the periphyton effect was stronger than the effect of grazer species identity. The seasonal increase in periphyton EPA% was reflected in the FA composition of grazer tissues, consistent with previous studies (Torres‐Ruiz et al. 2007; Guo et al. 2016 *a*). The observed correlations between grazer EPA and periphyton EPA could represent a combination of spatiotemporal variability in resources and a physiological need to fulfill EPA requirements in these grazers. These correlations match with the life cycles of the grazing mayflies we studied, i.e., *Baetis* sp. and *Ecdyonurus* sp. In summer, mayfly EPA was significantly correlated with periphyton EPA, probably because summer is the fast growing season for mayflies (Clifford et al. 1973; Humpesch 1979), and increasing assimilation of EPA can improve their somatic growth (Brett and Müller‐Navarra 1997). This suggests that grazers in summer may mainly act as “LC‐PUFA collectors.” Conversely, in fall, those mayfly grazers may allocate more EPA for reproduction or for overwintering (Clifford et al. 1973), partly accounting for the nonsignificant correlations between grazers and periphyton. They may mainly act as “LC‐PUFA integrators.” Furthermore, different patterns of grazer FA in response to periphyton FA in two seasons indicate the potential ability of those grazers to regulate their LC‐PUFA composition for physiological requirements. However, recent studies found that grazer somatic growth and PUFA patterns are, although in part taxa specific, likely mediated by dietary LC‐PUFA content (Guo et al. 2016 *a*). This is further confirmed by partial RDA results that the effect of periphyton FA on grazer FA profiles was stronger than that of species identity. Therefore, we suggest that grazers may utilize mixed feeding strategies for their EPA requirement, which mainly depends on periphyton FA variation across seasons.

Interestingly, although periphyton FA showed a limited effect on shredder FA, shredder EPA was still 6.9 times higher than in periphyton, suggesting shredders assimilate EPA very efficiently. It is well studied that shredders prefer feeding on conditioned rather than fresh leaves (Nolen and Pearson 1993; Pearson and Connolly 2000; Allan and Castillo 2007), and biofilms attached to leaf surfaces are a potential source of EPA (Torres‐Ruiz and Wehr 2010; Guo et al. 2016 *c*). However, in summer, conditioned leaves were not available in our streams, and it has been frequently reported in previous studies that shredders may face seasonal shortages of conditioned leaves during the late spring and summer period (Dobson and Hildrew 1992; Wallace et al. 1995). Their growth can be restricted in late summer by low‐food availability (Gee 1988). Therefore, shredders may act as “LC‐PUFA integrators,” selectively retaining limited dietary EPA or maintaining a quasi‐homoeostatic LC‐PUFA for their physiological requirements. Conversely, in fall, litter inputs provided shredders access to EPA by selectively feeding on EPA‐rich biofilms, which can enhance shredder growth (Franken et al. 2005; Guo et al. 2016 *c*). The strong correlations between shredder EPA and periphyton EPA in fall indicated that shredders may actively feed on leaves with EPA‐rich biofilms and may preferentially assimilate EPA over other FA. In contrast to grazers, the significant relationship between shredder EPA and periphyton EPA was observed in fall rather than in summer, as shredders capitalize on the fall litter inputs. Thus, it is possible that shredders may selectively assimilate more EPA over other FA (collectors) in fall when high‐quality food is abundant, whereas they may act as “LC‐PUFA integrators” in summer due to the limited access to dietary EPA.

Compared with grazers and shredders, predator FA composition was less affected by periphyton FA and predator species identity. Recent isotope studies suggest that algal resources are even more important for secondary than primary consumers in benthic habitats (Bunn et al. 2013; Lau et al. 2014). However, in our study, no significant relationships between the EPA content of grazers and shredders with predators were observed, and periphyton FA effect on predators was low, suggesting that predators in our study may actively control their EPA with respect to different quality foods. Predator FA profiles have been found to be distinct at low taxonomic resolution, among classes or higher levels (Lau et al. 2012), which indicates the low effect of species identity and their potential ability to maintain a quasi‐homoeostatic FA profile regardless of different food quality. Furthermore, although the periphyton FA effect on predators was low, EPA% is increasingly retained along the trophic gradient from periphyton to predators via primary consumers (grazers and shredders), confirming the importance of EPA in stream ecosystems for efficient trophic transfer to upper trophic levels (Gladyshev et al. 2011; Lau et al. 2012; Guo et al. 2017).

Our study suggests that macroinvertebrates can adapt to seasonally or spatially available dietary LC‐PUFA, in particular EPA. Seasonal and spatial environmental factors showed less effect on macroinvertebrate FA compared with the effect of periphyton FA and invertebrate species identity. Macroinvertebrates may be able to feed selectively on, assimilate, and/or actively control their LC‐PUFA distribution in the face of changing environmental conditions. Although the proposed view of LC‐PUFA collectors/integrators is restricted to stream invertebrates, it is possible that this holds also true for other aquatic consumers, including zooplankton and higher consumers such as fish in lotic and lentic freshwater ecosystems, and possibly in marine ecosystems since these aquatic consumers may all experience changes in resource LC‐PUFA availability. Future research is required to better comprehend the feeding strategies for the acquisition of high‐quality food sources in aquatic consumers.

## Conflict of Interest

None declared.

## Supporting information

Supporting Information Appendix Table A1

Supporting Information Appendix Figures
