# Supplementary material for: Feeding strategies for the acquisition of high‐quality food sources in stream macroinvertebrates: Collecting, integrating, and mixed feeding
Source: Limnol Oceanogr. 2018 May 6;63(5):1964–78. doi: 10.1002/lno.10818 (PMC6283091; doi:10.1002/lno.10818)
Supplement: Supplementary file 2 — Supporting Information Appendix Figures [file LNO-63-1964-s002.pdf]

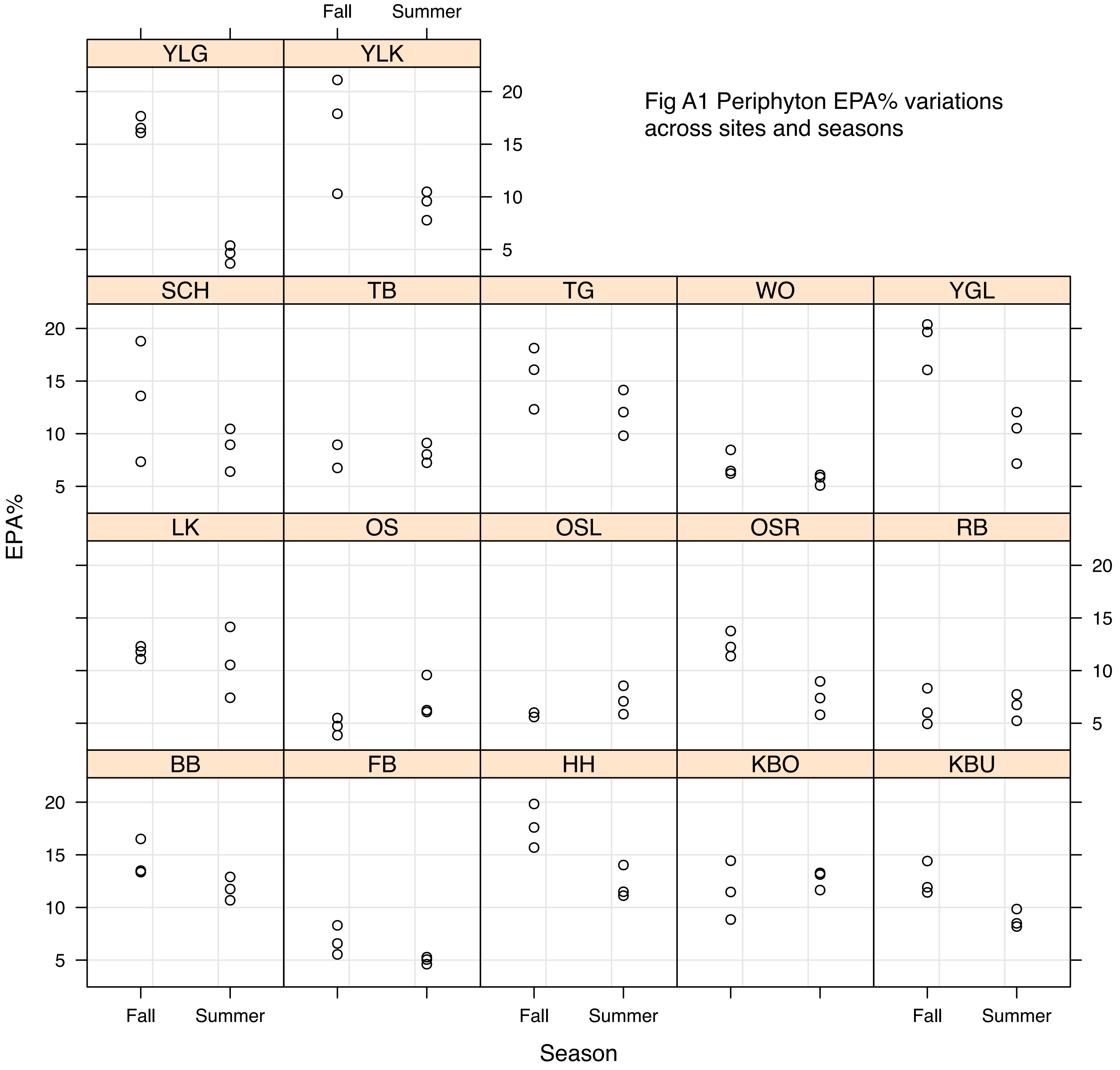
Fig A1 Periphyton EPA% variations
across sites and seasons
YLG
YLK
SCH
TB
TG
WO
YGL
LK
OS
OSL
OSR
RB
BB
FB
HH
KBO
KBU
EPA%
Season
Fall
Summer
5
10
15
20

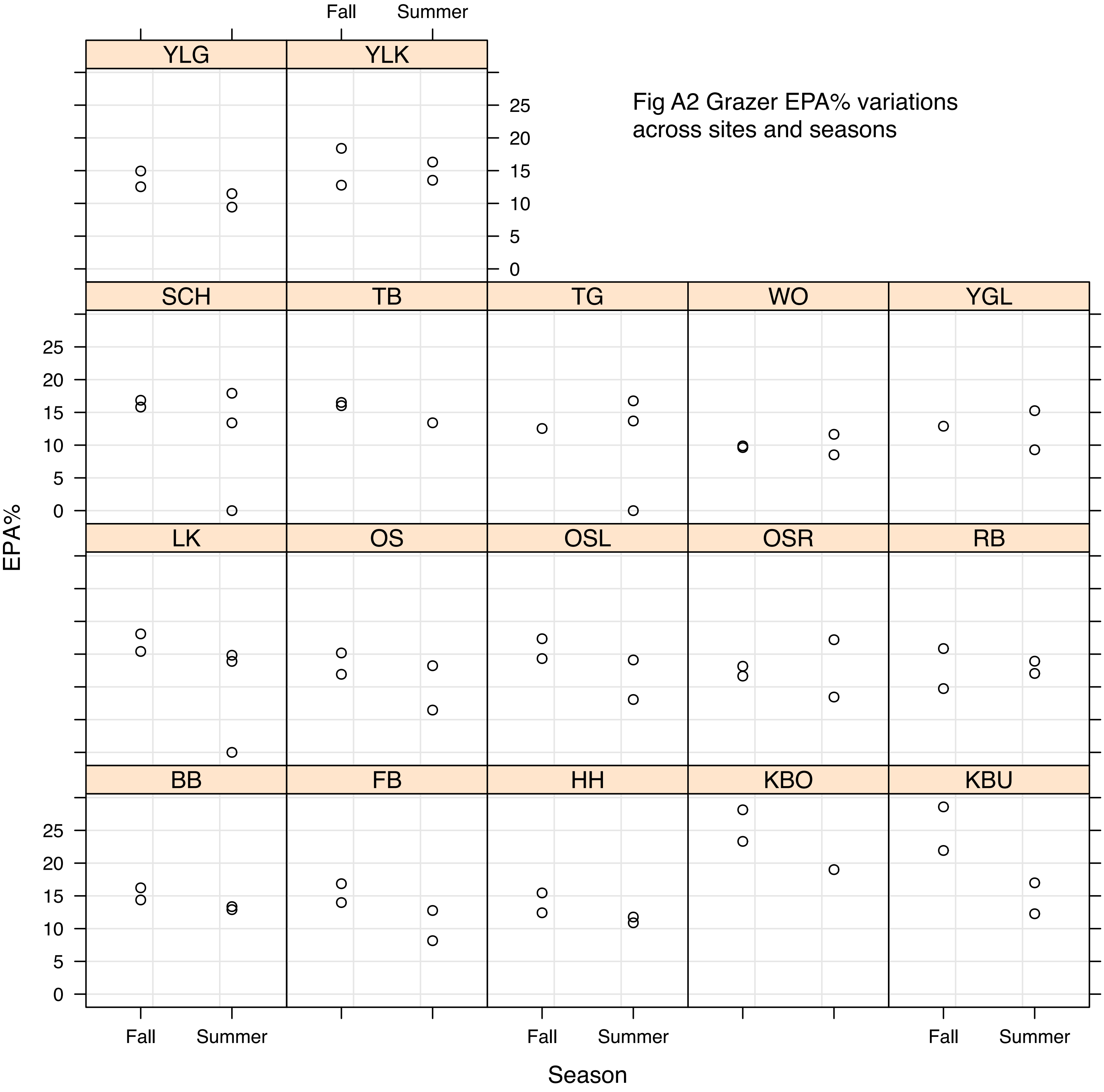

Fig A2 Grazer EPA% variations across sites and seasons

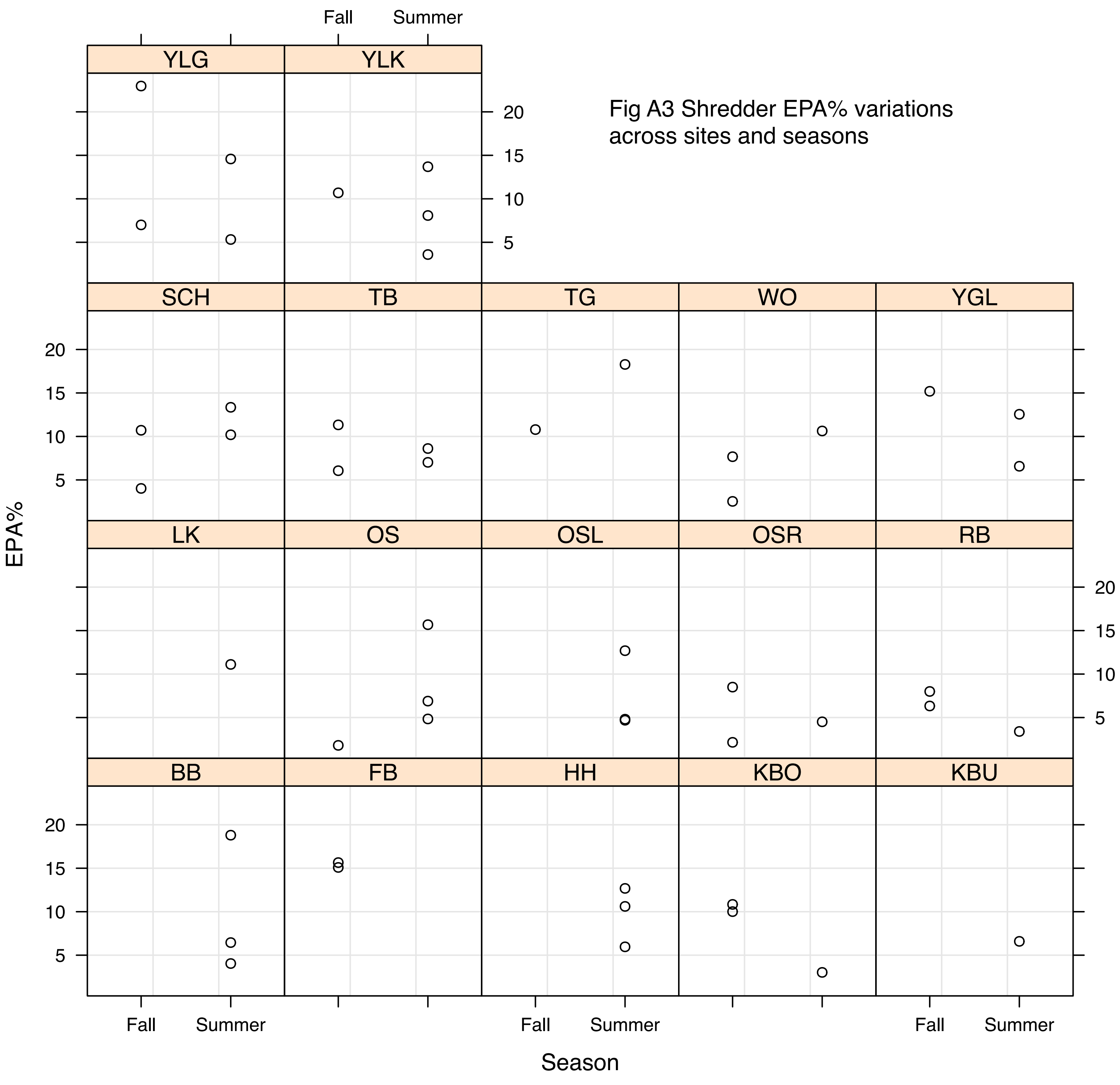

Fig A3 Shredder EPA% variations across sites and seasons
